# The influence of marital status on stage at diagnosis and survival of patients with colorectal cancer

**DOI:** 10.18632/oncotarget.3129

**Published:** 2015-02-06

**Authors:** Qingguo Li, Lu Gan, Lei Liang, Xinxiang Li, Sanjun Cai

**Affiliations:** ^1^ Department of Colorectal Surgery, Fudan University Shanghai Cancer Center, Shanghai, People's Republic of China; ^2^ Department of Medical Oncology, Fudan University Shanghai Cancer Center, Shanghai, People's Republic of China; ^3^ Department of Oncology, Shanghai Medical College, Fudan University, Shanghai, People's Republic of China

**Keywords:** Colorectal cancer, marital status, SEER, survival analysis

## Abstract

Marital status was found to be an independent prognostic factor for survival in various cancer types, but it hasn’t been fully studied in colorectal cancer (CRC). The Surveillance, Epidemiology and End Results database was used to compare survival outcomes with marital status in each stage. In total, 112, 776 eligible patients were identified. Patients in the widowed group were more frequently elderly women, more common of colon cancer, and more stage I/II in tumor stage (*P* < 0.001), but the surgery rate was comparable to that for the married group (94.72% VS 94.10%). Married CRC patients had better 5year cause-specific survival (CSS) than those unmarried (*P* < 0.05). Further analysis showed that widowed patients always presented the lowest CSS compared with that of other’ group. Widowed patients had 5% reduction 5-year CSS compared with married patients at stage I (94.8% *vs* 89.8%, *P* < 0.001), 9.4% reduction at stage II (85.9% *vs* 76.5%, *P* < 0.001), 16.7% reduction at stage III (70.6% *vs* 53.9%, *P* < 0.001) and 6.2% reduction at stage IV(14.4% *VS* 8.2%, *P* < 0.001). These results showed that unmarried patients were at greater risk of cancer specific mortality. Despite favorable clinicpathological characteristics, widowed patients were at highest risk of death compared with other groups.

## INTRODUCTION

Married individuals enjoy longer overall survival and lower mortality for many major causes of death compared with those who have never married, separated, widowed, or divorced [1–3]. Extensive research has shown that marital status is an independent prognostic factor of survival in various cancer types [4–9]. In a larger population-based study on data from the Surveillance, Epidemiology and End Results (SEER) database indicated that unmarried patients are at significantly higher risk of presentation with metastatic cancer, undertreatment, and death resulting from their cancer in ten leading causes of cancer-related death [4]. Similarly, Johansen et al. [8] and Wang et al. [9] reported that patients with colon cancer who were married at the date of diagnosis survived significantly longer than those who had never been married. However, the study by Johansen et al. compared survival outcomes of married and unmarried individuals without differentiating among single, divorced and widowed status. Additionally, marital statuses in the population, stage at presentation, mortality, as well as therapy options, have changed in more recent years. This change may be related to possible increases or decreases in the proportion of married and unmarried individuals and their effect on cause-specific survival (CSS) [10, 11]. Moreover, two reported reasons of poor survival among unmarried patients were delayed diagnosis and undertreatment. If this were true, marital status may have no effect on early CRC, because these patients do not require adjuvant therapy. Given that CRC is one of the most common malignancies and is ranked as the third leading cause of cancer-related deaths in the USA [12] and marriage is an important aspect of adult life, it is important to explore the relationship between marital status and CRC and the potential underlying mechanisms. In this study, we used data from the SEER cancer-registry program of individuals diagnosed between 2004 and 2008 to explore in detail what aspects of marital status affects cancer survival. Our hypothesis was that the unmarried subgroup of CRC patients may differ in terms of survival outcomes.

## RESULTS

### Patient baseline characteristics

A total of 112, 776 eligible patients were identified during the 4-year study period, including 57, 921 male and 54, 855 female patients. Of these, 62, 255 (55.20%) were married, 21, 279 (18.87%) were widowed, and 15, 043 (13.34%) had never married. The 1, 044 (0.93%) individuals who were separated and 10, 155 (9.00%) who were divorced were grouped together in the divorced/separated group in our study [9]. Patients in the widowed group had the highest proportion of women, more common of colon cancer, more prevalence of elderly patients (≥60 years), and more tumor at stage I/II, all of which were statistically significant (*P* < 0.001). The rate of surgery performed was comparable between the married and widowed groups (94.72% *vs* 94.10%), but higher than that in the never married (91.31%) and divorced/separated (92.47%) group. Patient demographics and pathological features are summarized in Table 1.

**Table 1:** Baseline demographic and tumor characteristics of patients in SEER database

Characteristic	Total	Married	Widowed	Never married	Divorced/Separated	*P* value
	(*n* = 112776)	(*n* = 62255) *N* (%)	(*n* = 21279) *N* (%)	(*n* = 15043) *N* (%)	(*n* = 11199) *N* (%)	
Sex						< 0.001
male	57921	39890(61.1)	4539(21.3)	8212(54.6)	5280(47.1)	
female	54855	25365(38.9)	16740(78.7)	6831(45.4)	5919(52.9)	
Primary Site						< 0.001
Colon	94496	54139(83.0)	18913(88.9)	12396(82.4)	9048(80.8)	
Rectum	18280	11116(17.0)	2366(11.1)	2647(17.6)	2151(19.2)	
Age						< 0.001
<60	35126	22522(34.5)	874(4.1)	7415(49.3)	4315(38.5)	
≥60	77650	42733(65.5)	20405(95.9)	7628(50.7)	6884(61.5)	
Race						< 0.001
White	90608	53736(82.3)	17630(82.9)	10557(70.2)	8685(77.6)	
Black	12729	5329(8.2)	2204(10.4)	3333(22.2)	1863(16.6)	
Other^*^	9136	6001(9.2)	1409(6.6)	1104(7.3)	622(5.6)	
Unknown	303	189(0.3)	36(0.2)	49(0.3)	29(0.3)	
Pathological grading						< 0.001
High/Moderate	83291	48680(70.6)	15418(72.5)	11079(73.6)	8114(72.5)	
Poor/Anaplastic	21969	12368(19.0)	4588(21.6)	2773(18.4)	2240(20.0)	
Unknown	7516	4207(6.4)	1273(6)	1191(7.9)	845(7.5)	
Histotype						< 0.001
Adenocarcinoma	102078	59181(90.7)	19149(90.0)	13580(90.3)	10168(90.8)	
Mucinous cell	9309	5243(8)	1919(9.0)	1247(8.3)	900(8.0))	
Signet ring cell	1389	831(1.3)	211(1.0)	216(1.4)	131(1.2)	
TNM stage						< 0.001
I	23689	14785(22.7)	4251(20.0)	2562(17.0)	2091(18.7)	
II	32877	18387(28.2)	7084(33.3)	4223(28.1)	3183(28.4)	
III	31746	18575(28.5)	5861(27.5)	4171(27.7)	3139(28.0)	
IV	24464	13508(20.7)	4083(19.2)	4087(27.2)	2786(24.9)	

* Other includes American Indian/Alaska native, Asian/Pacific Islander, etc.

### Effect of marital status on CSS in the SEER database

**Figure 1:**
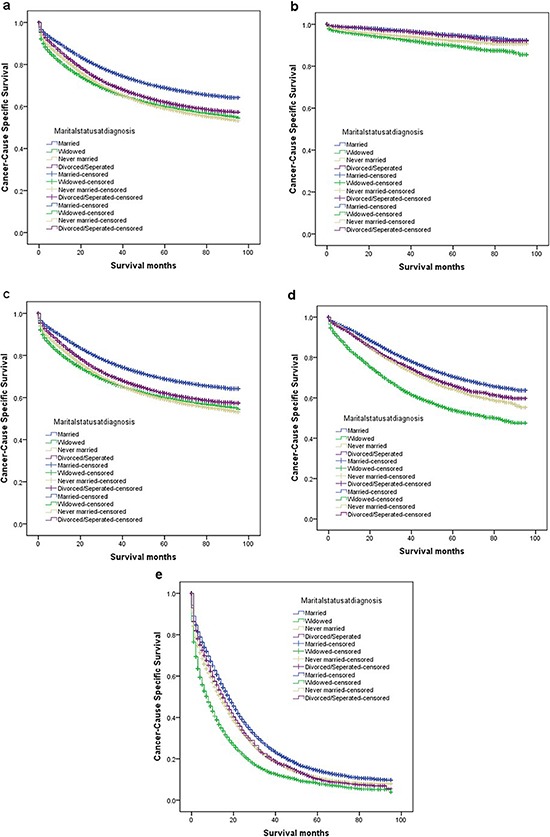
Survival curves in colorectal patients according to marital status **(a)** stage I-IV: χ^2^ = 1096.367, *P* < 0.001; **(b)** stage I: χ^2^ = 154.618, *P* < 0.001; **(c)** stage II: χ^2^ = 346.777, *P* < 0.001; **(d)** stage III: χ^2^ = 646.624, *P* < 0.001; **(e)** stage IV: χ^2^ = 602.869, *P* < 0.001.

The overall 5-year CSS was 68.9% in the married group, 60.0% in the widowed group, 59.2% in the never married group, and 60.0% in the divorced/separated group, which were all significantly different according to the univariate log-rank test (*P* < 0.001) (Figure 1). Additionally, elderly patients (*P* < 0.001), male sex (*P* < 0.001), black ethnicity (*P* < 0.001), poor or undifferentiated tumor grade (*P* < 0.001), mucinous or signet-ring cancer (*P* < 0.001), higher American Joint Committee on Cancer (AJCC) stage (*P* < 0.001), and no surgery (*P* < 0.001) were identified as significant risk factors for poor survival on univariate analysis (Table 2). When multivariate analysis with Cox regression was performed, all seven variables were validated as independent prognostic factors. These included age (≥60 years, hazard ratio (HR) 1.522, 95% confidence interval (CI) 1.487–1.558), ethnicity(black, HR 1.182, 95%CI 1.147–1.218; others, HR 0.899, 95% CI 0.865–0.935), pathological grading(poor or undifferentiated tumor, HR 1.457, 95% CI 1.422–1.492; unknown, HR 1.689, 95% CI 1.623–1.739), histologic type (mucinous/signet ring cell, HR 1.091, 95% CI 1.056–1.127), AJCC stage(stage II, HR 2.723, 95% CI 2.570–2.885; stage III, HR 5.897, 95% CI 5.581–6.231; stage IV, HR 30.707, 95% CI 29.101–32.401), surgery (no surgery performed, HR 2.123, 95%CI 2.053–2.196), marital status(widowed, HR 1.485, 95%CI 1.445–1.526; never married, HR 1.307, 95%CI 1.269–1.347; divorced/separated, HR1.181, 95% CI 1.142–1.222).

**Table 2:** Univariate and multivariate survival analysis for evaluating the influence of marital status on colorectal cause-specific survival in SEER database

Variable	5-year CCS	Univariate analysis		Multivariate analysis	
		Log rank χ^2^ test	*P*	HR(95%CI)	*P*
**Primary Site**		0.944	0.331		NI
Colon	65.5%				
Rectum	64.3%				
**Sex**		1.768	0.184		NI
Male	64.9%				
Female	65.7%				
**Age**		195.03	<0.001		<0.001
<60	67.4%			Reference	
≥60	64.3%			1.522(1.487–1.558)	
**Race**		406.282	<0.001		
White	66.1%			Reference	<0.001
Black	57.3%			1.182(1.147–1.218)	
Other^*^	68.2%			0.899(0.865–0.935)	
**Grade**		5557.256	<0.001		<0.001
High/Moderate	70.6%			Reference	
Poor/Anaplastic	52.3%			1.457(1.422–1.492)	
Unknown	43.6%			1.689(1.623–1.739)	
**Histotype**		284.69	<0.001		0.571
Adenocarcinoma	66.1%			Reference	
Mucinous/signet ring cell	57.9%			1.009(0.977–1.043)	
**TNM Stage**		62866.96	<0.001		<0.001
I	93.6%			Reference	
II	82.8%			2.723 (2.570–2.885)	
III	66.4%			5.897(5.581–6.231)	
IV	12.3%			30.707(29.101–32.401)	
**Marital Status**		1096.367	<0.001		<0.001
Married	68.9%			Reference	
Windowed	60.0%			1.485(1.445–1.526)	
Never married	59.2%			1.307(1.269–1.347)	
Divorced/Separated	62.0%			1.181(1.142–1.222)	

* Other includes American Indian/Alaska native, Asian/Pacific Islander, and unknown.

NI: not included in the multivariate survival analysis.

### Subgroup analysis for evaluating the effect of marital status according to AJCC stage

One reason previously reported of poor prognosis of unmarried patients is delayed diagnosis. If this is true, once the tumor is diagnosed, marital status should not affect CSS. Another reason reported is undertreatment. If so, patients at an early stage should not be affect by marital status because they do not require adjunctive therapy. Therefore, we made further analysis of the effects of marital status on survival in each tumor stage. We observed three interesting findings. First, marital status was an independent prognostic factor in each tumor stage both in univariate and multivariate analysis (*P* < 0.001). Second, patients in the widowed group always had the lowest survival rate when compared with patients in the other groups. Widowed patients had 5% reduction in 5-year CSS compared with married patients at stage I (94.8% *vs* 89.8%, *P* < 0.001), 9.4% reduction at stage II (85.9% *vs* 76.5%, *P* < 0.001), 16.7% reduction at stage III (70.6% *vs* 53.9%, *P* < 0.001) and 6.2% reduction at stage IV(14.4% *vs* 8.2%, *P* < 0.001). Third, the difference between the divorced/separated and never married group was not apparent. Compared with patients in the never married group, patients in the divorced/separated group at stage I-III had an increase of 1.3–2.4% in 5-year CSS and, a 0.6% decreased in survival at stage IV Table 3.

**Table 3:** Univariate and multivariate analysis of marital status on colorectal cancer cause specific survival based on different cancer stage

Variable	5-year CCS	Univariate analysis		Multivariate analysis	
		Log rank χ^2^ test	*P*	HR(95% CI)	*P*
**TNM Stage**					
**Stage I**					
**Marital status**		154.618	<0.001		
Married	94.8%			Reference	
Widowed	89.8%			1.722(1.522–1.947)	<0.001
Never married	92.1%			1.592(1.355–1.872)	<0.001
Divorced/Separated	94.5%			1.077(0.883–1.313)	0.463
**Stage II**					
**Marital status**		346.77	<0.001		
Married	85.9%			Reference	
Widowed	76.5%			1.532(1.434–1.637)	<0.001
Never married	80.5%			1.555(1.434–1.688)	<0.001
Divorced/separated	81.8%			1.314(1.197–1.441)	<0.001
**Stage III**					
**Marital status**		646.624	<0.001		
Married	70.6%			Reference	
Widowed	53.9%			1.575(1.498–1.656)	<0.001
Never married	64.4%			1.332(1.254–1.414)	<0.001
Divorced/separated	66.1%			1.182(1.105–1.266)	<0.001
**Stage IV**					
**Marital status**		602.869	<0.001		
Married	14.4%			Reference	
Widowed	8.2%			1.400(1.345–1.456)	<0.001
Never married	10.9%			1.228(1.180–1.277)	<0.001
Divorced/separated	10.3%			1.151(1.101–1.204)	<0.001

*P*-values refer to comparisons between two groups and were adjusted for age, race, pathological grading, and tumor histologic type as covariates.

NI: not included in the multivariate survival analysis.

## DISCUSSION

Although the impact of marriage on CRC survival has been studied using both SEER as well as other country-specific cancer databases [4, 8, 9], no research has been performed on stage by stage comparisons of the effects of marital status on patient survival or focused on the heterogeneity of unmarried patients. Our study indicated that marital status was an independent prognostic factor in each TNM stage. Additionally, although patients in the widowed group had the highest percentage of early tumor stage (I/II), they had the worst survival when compared with those in the other group.

One hypothesis to explain the unfavorable prognosis of unmarried individuals is undertreatment. However, in any current guideline for CRC clinical practice, adjunctive therapy is not recommended for patients in stages I patients and II without risk factors. We found that patients in the widowed group still had a disadvantage of 5% in stage I and 9.4% in stage II regarding the 5-year CSS compared with those in the married group. Moreover, the rate of surgical resection was comparable between both the married and widowed groups. None of these finding could be explained by the hypothesis of undertreatment. Johansen et al, also found that the observed effect of marital status could not be attributed to treatment options for accessing to health care services is provided free in Denmark during the period under study [8]. Interestingly, delayed diagnosis was considered as another reason for poor prognosis in unmarried patients [4, 13, 14]. However, in our study group, the percentage of patients with CRC in stage I and II CRC patients was highest in the widowed group with 53.3% compared with 50.9%, 45.1%, and 47.1% in the married, never married, and divorced/separated group, respectively. Obviously, this result is paradoxical given the poor survival outcomes in the widowed group.

Our data revealed that unmarried patients have a survival disadvantage that persists in each TNM stage. The relationship between marital status and survival can be explained hypothetically by psychosocial factors that are independent of tumor characteristics and extent of treatment. It has been proposed that decreased psychosocial support and psychological stress alter immune function and contribute to tumor progression and mortality [15–17]. Levy et al. reported that a perceived lack of social support was associated with lower activity of natural killer cells [18]. Chronic stress may elicit prolonged secretion of cortisol [19], that triggers a counterregulatory response of white blood cells by downregulating their cortisol receptors. This downregulation, in turn, reduces the cell’ capacity to respond to anti-inflammatory signals and allows cytokine-mediated inflammatory processes to flourish [20], which have been validated as poor prognostic factors in CRC [21, 22]. Conversely, cortisol levels seem to be lower in patients with cancer who have adequate support networks, and diurnal cortisol patterns have been linked with natural-killer cell count and survival in patients with cancer [23, 24]. Additionally, depression and quality of life have been related to VEGF, which may stimulate endothelial cell migration, proliferation and proteolytic activity [25], in CRC [26]. Additionally, some other neuroendocrine mediators and cytokines present in depression and stress are linked with cancer metastasis [17]. Unrecognized clinical depression is strongly associated with poor adherence to medical treatment [27]. Meta-analyses of the impact of depression on cancer mortality confirm increased death rates between 19% and 39% [28, 29]. The loss of social support or the inability to cope with stress in the widowed groups seems very apparent, and may lead to excess mortality [30].

Age is another factor that should be considered fully. The proportion of elderly patients (≥60 years) in the widowed group was extremely high (95.9%), which may be another reason for extremely poor survival in this group. Age itself is a prognostic factor in CRC [31, 32] that can be explained by aging impaired immune response, increased oxidative stress, shortening of telomeres, accumulation of senescent cells [33, 34].

This study adds to the current knowledge by answering more in-depth research questions about marital status and prognosis through the analysis of data from the large population-based SEER database. However, it had several potential limitations. First, the SEER database only provide the marital status at diagnosis. Whether the marital status changed after diagnosis is unknown, and this change may also affect patient’ survival. Second, SEER database lacks information of education, income status, insurance status, socioeconomic status and quality of marriage, which might confound the explanation of the disparity in survival between marital groups. For example, marital distress has long term immune consequences and enhances the risk of a variety of health problems [35]. Third, information on therapy options (surgical resection or palliative therapy), subsequent therapy, co-morbidities and recurrence is also lacking. Fourth, we hypothesized that psychosocial factors may be the main reasons for poor survival of unmarried patients, but we performed a retrospectively analysis using a public database, and we could not performed psychological tests to validate our hypothesis.

Despite these potential limitations, our study results confirmed that unmarried patients are at greater risk of cancer-specific mortality. Moreover, we indicated that the unmarried patients groups was heterogeneous, and the widowed patients were always at the highest risk of death of cancer than those in other groups. Psychosocial factors may be the main reasons for poor survival outcomes in unmarried patients. Physicians caring for unmarried patients with CRC, especially those who are widowed should be aware of their poorer outcomes. Additionally, social support systems should provide closer cares and interventions for these patients to help reduce the significant survival differences between married and unmarried patients with CRC cancer.

## METHODS

### Patient selection in the SEER database

The SEER Cancer Statistics Review (http://seer.cancer.gov/data/citation.html), a report on the most recent cancer incidence, mortality, survival, prevalence, and lifetime risk statistics, is published annually by the Data Analysis and Interpretation Branch of the National Cancer Institute, USA. The current SEER database consists of 17 population-based cancer registries that represent approximately 28% of the population in the US. It contains no identifiers and is widely used for studies of the relationship between marital status and survival outcomes of patients with cancer [4, 5, 7, 9–11].

Using the SEER-stat software (SEER*Stat 8.1.5), we searched for patients diagnosed between 2004 and 2008 with single primary CRC and a known marital status. Histological type were limited to adenocarcinoma (8150/3, 8210/3, 8261/3, 8263/3), mucinous adenocarcinoma (8480/3), and signet ring cell carcinoma (8490/3). Patients were excluded if age at diagnosis was less than 18 years, they had undefined TNM stage, had more than one primary cancer but the CRC wasn’t the first one, had unknown cause of death or unknown survival months.

### Statistical analysis

Age, sex, ethnicity, extension of primary tumor invasion, lymph nodes status, histological grade, survival time, and CSS were extracted from the SEER database. All cases were restaged according to the criteria described in the AJCC Cancer Staging Manual (7th edition, 2010). Within the SEER database, marital status of the patient is recorded at the time of diagnosis. Marital status is coded as married, divorced, widowed, separated, and never married. Individuals in the separated and divorced group were clustered together as the divorced/separated group in this study.

Patient baseline characteristics were compared with the χ^2^ test, as appropriate. The rate of CRC death was compared between groups using the Kaplan–Meier method. Multivariable Cox regression models were built for analysis of risk factors for survival outcomes. The primary endpoint of this study was CSS, which was calculated from the date of diagnosis to the date of cancer specific death. Deaths attributed to CRC were treated as events and deaths from other causes were treated as censored observations. All of statistical analyses were performed using the statistical software package SPSS for Windows, version 17 (SPSS Inc, Chicago, IL, USA). Statistical significance was set at two-sided *P* < 0.05.
